# Single lesion multibacillary leprosy, a treatment enigma: a case report

**DOI:** 10.1186/1752-1947-3-8

**Published:** 2009-01-13

**Authors:** Bishwa R Sapkota, Kapil D Neupane, Ram K Maharjan

**Affiliations:** 1 Mycobacterial Research Laboratory, Anandaban Hospital, GPO Box 151, Kathmandu, Nepal

## Abstract

**Introduction:**

Leprosy exhibits a wide spectrum of presentation, varying from the tuberculoid to the lepromatous pole, with immunologically unstable borderline forms in-between, depending upon the immune status of the individual. The clinical system of classification for the purpose of treatment includes the number of skin lesions and nerves involved as the basis for classifying the patients into multibacillary and paucibacillary.

**Case presentation:**

A 20-year-old man belonging to a moderately endemic leprosy area in the Terai region of Nepal reported a large single, hypopigmented, well defined anaesthetic lesion on his left thigh extending to his knee which had been present for 2 years. There was no other nerve involvement. Clinical diagnosis was tuberculoid leprosy and immunological lateral flow test for anti-Phenolic glycolipid-I antibody was positive. Six months of paucibacillary multidrug treatment was advised immediately. However, the patient was reclassified as multibacillary on the basis of a positive skin smear and appropriate treatment of 24 months multibacillary multidrug regimen was commenced after only 1 week. Slit skin smear examination for *Mycobacterium leprae *from the lesion revealed a bacterial index of 4+ while it was negative from the routine sites. Histopathological examination from skin biopsy of the lesion further supported the bacterial index of the lesion granuloma which was 2+ and the patient was diagnosed as borderline tuberculoid. Bacteriological, histological, and immunological findings of this patient were borderline tuberculoid leprosy and he should have been treated with multibacillary regimen from the beginning. Five months after commencement of treatment, the patient developed a leprae reaction of Type 1 or reversal reaction with some nerve function impairment and enlargement of the lateral popliteal nerve of the left leg. This reversal reaction was managed by standard oral prednisolone whilst continuing the multibacillary multidrug regimen.

**Conclusion:**

This case illustrates and emphasizes the importance of slit-skin smear and biopsy as routine in all new cases to help differentiate multibacillary from paucibacillary for correct treatment. It further suggests that there are factors yet undetermined which play a significant role in determining the host response to *M. leprae *which is a remaining challenge in this disease.

## Introduction

Leprosy is a chronic infectious disease caused by *Mycobacterium leprae *and is an ancient scourge that still affects ≥ 4 persons per 10,000 in Brazil, India, Madagascar, Mozambique and Nepal. Globally, Nepal is among the six major endemic countries which account for 23% of all new cases detected during 2005 and 24% of registered cases at the beginning of 2006 [1,2]. Leprosy exhibits a wide spectrum of presentation, varying from the tuberculoid to the lepromatous pole, with the immunologically unstable borderline forms in-between, depending upon the immune status of the individual. At the lepromatous pole, the patients lack effective cell-mediated immunity to *M. leprae *and bacilli proliferate, while at the tuberculoid pole, the patients have cell mediated immunity towards *M. leprae *and there is elimination of mycobacteria.

## Case presentation

**Figure 1:**
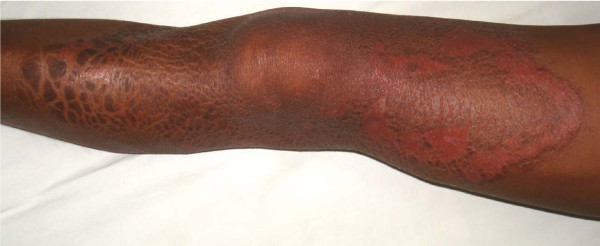
**Initial solitary lesion plaque after 5 months of multibacillary multidrug treatment**. Reversal reaction: 5 months after initial presentation and commencement of multibacillary multidrug treatment.

A 20-year-old man belonging to a moderately endemic leprosy area in the Terai region of Nepal presented in our clinic as a referred patient from a teaching hospital with the history of 2 years. On clinical examination, a solitary hypopigmented, anaesthetic lesion was present on his left thigh extending to his knee. The surface of the lesion was dry and it had a length of approximately 15 cm with raised edge as shown in Figure 1. No other lesions were noted over the entire body. There was no peripheral nerve thickening. No history of contact with leprosy could be elicited. Furthermore, the rapid lateral flow test (supplied by Linda Oskam, KIT Biomedical Research, The Netherlands and Ray Cho, Yonsei University, Korea) for anti-Phenolic glycolipid-I (PGL-I) antibody of *M. leprae *was positive. Blood count was normal and the standard nerve function measurements by voluntary muscle testing and sensory test (VMT/ST) of the hand and feet were within normal limits. His slit skin smear and biopsy were sent for laboratory investigations.

His clinical presentation was consistent with tuberculoid leprosy (TT) and paucibacillary (PB) multidrug treatment (MDT) was initiated immediately comprising 600 mg of Rifampicin once a month and 100 mg of Dapsone daily. The patient was under PB MDT for a week; however, once the slit skin smear report was obtained, this prompted the clinician to change treatment from 6 months PB to 24 months multibacillary (MB) MDT regimen comprising Rifampicin 600 mg once a month, Clofazimine 300 mg once a month followed by 50 mg daily and Dapsone 100 mg daily.

**Figure 2:**
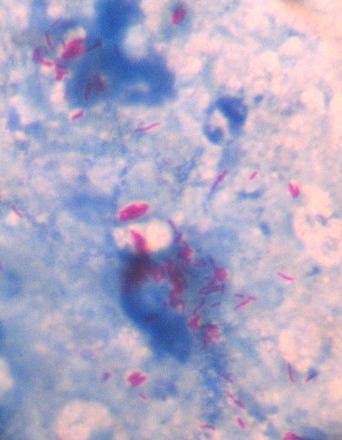
**Microscopic observation of acid fast bacilli (red rods) in the slit skin smear fluid from the solitary lesion in the initial presentation of the patient**.

Slit skin smear examination for *M. leprae *from the lesion revealed a Bacterial Index (BI) of 4+ (10–100 acid fast bacilli per oil immersion microscopic field on Ridley's logarithmic scale) as shown in Figure 2 while it was negative from the routine sites of the ear lobes, thigh and arm. The Morphological Index was 0%. On histopathological examination, the biopsy from the lesion revealed skin and subcutaneous tissue. There was an epidermal atrophy with mild orthokeratosis. Small aggregates of histocytes and lymphocytes were seen around vessels and skin adnexa and in and around nerves. The granuloma fraction was about 5%. Solid acid fast bacilli were seen in some of the granulomas, singly and in small clusters of 3 to 5 bacilli; the BI of the granuloma was 2+. Based on the smear results and histopathological report, the patient was classified as borderline tuberculoid (BT) in the spectrum of Ridley and Jopling's classification [3].

Five months after commencement of MB MDT, the patient developed a Type 1 or reversal reaction. This time, there was palpable enlargement of a nerve and evidence of nerve function impairment was observed. Again, we assessed the patient for clinical, bacteriological and immunological investigations. Clinically, the patient still had the same single lesion. Bacteriologically, the routine sites were still negative and the BI of the lesion was 1+. The lateral flow for anti-PGL-I antibody was positive with lesser intensity than the test done on his first visit indicating a lower bacterial load. The standard nerve function measurements by VMT/ST were normal on both feet and hands but some weakness was observed on the left foot which concurs with enlargement of the left lateral popliteal nerve.

Blood count was normal at this time and a whole blood interferon gamma (IFN-γ) test was also performed to measure the cell mediated immune responses towards *Mycobacterium tuberculosis *purified protein derivative (MTB PPD), *M. leprae *soluble antigen devoid of lipoarabinomannan (MLSA-LAM) and *M. leprae *cell wall antigen (MLCwA), which were free of endotoxin and *M. leprae *lipoarabinomannan, supplied by Professor Patrick J. Brennan, Colorado State University, Fort Collins under NIH contract NO1-AI-25469. These were tested by QuantiFERON-CMI (Cellestis, Australia). IFN-γ production levels were generated in response to stimulation with Cellestis mitogen but no detectable levels of IFN-γ were obtained against these antigens in our patient.

The reversal reaction was treated with 12 weeks of oral prednisolone in a tapering dose and MDT was continued unaltered during this period. Initially, the prednisolone was prescribed at a dose of 40 mg per day for a 2-week period, then decreased to 30 mg per day for 2 weeks, 20 mg per day for 2 weeks, 15 mg per day for 2 weeks, 10 mg per day for another 2-week period and finally 5 mg per day for 2 weeks.

## Discussion

A solitary lesion with a high bacterial count is a rare occurrence and has been reported as an unusual presentation [4-8]. The clinical presentation of our patient was consistent with TT, but based on the evidence from histopathology and bacteriology, the patient was shown to be borderline tuberculoid (BT). Five months after the commencement of MB MDT, he developed a reversal reaction, which is due to delayed-type hypersensitivity and an increase cell-mediated immunity to *M. leprae *antigens resulting in localized tissue and nerve damage. Several studies have reported higher levels of IFN-γ in BT leprosy in Type 1 reaction; however, some studies have reported a lack of demonstrable consistency in relation to cytokine levels and reversal reactions [9].

Here, no IFN-γ was detected against the *M. leprae *fraction antigens from this patient at this time point. He responded well to oral prednisolone. Although, we could not establish a clear correlation between the IFN-γ response and the type of leprosy, using these antigens, a significant difference was shown between healthy leprosy contacts and unexposed endemic healthy controls in a Nepali population in the production of IFN-γ [10].

Clinically, this case appeared as TT leprosy. But the skin smear examination from the lesion showed an overall moderately high BI at the lesion and the histopathological examination confirmed that this was a lesion of BT leprosy. This clinical and histopathological discrepancy has been reported earlier [4,11,12]. This case emphasizes the importance of skin smears and the histopathological identification and classification of all patients with unusual lesions. Without such information, this patient would have been considered tuberculoid leprosy and would have received the continuous treatment designed for PB patients, which may not have been adequate.

Furthermore, the rapid lateral flow test for anti-Phenolic glycolipid-I (PGL-I) antibody of *M. leprae *was highly positive at the beginning and moderately positive at the follow-up. In one study, a lateral flow assay correctly diagnosed 97.4% MB leprosy patients, with a specificity of 86.2% [13]. Detection of anti-PGL-I antibodies may thus be an indicator of leprosy development and a useful complementary tool in diagnosing MB leprosy in support of clinical examination to direct the correct treatment regimen. Consequently, this evidence further supports the view that this patient should have been treated by MB MDT regimen from the beginning.

It is not always routinely possible to perform a histopathological examination, but skin smear from selective sites, which is a relatively minor procedure, should always be done in patients with one or a few skin patches to help in differentiating MB from PB cases. This is all the more important in a period when the disease is under control, and patients reporting with early lesions and advanced disease have become a rarity. This case is a rare case of localized BT leprosy, whereas reports of localized lepromatous leprosy involving the extremities have been reported earlier [12].

The WHO has recommended that skin smears are not a prerequisite for starting a patient on MDT. The clinical system of classification which has been recommended for the purpose of treatment includes the use of numbers of skin lesions and nerves involved as the basis for classifying patients into MB and PB. If in doubt, the WHO further recommends that the patient should be treated with MB regimen [1].

This case further highlights the importance of performing skin smears as a routine in all patients to differentiate MB from PB disease. In the light of this case and previous reports [4,5,14,15] of even lepromatous leprosy presenting as a single skin lesion, field workers, including both medical and paramedical workers should carefully perform and interpret slit-skin smears from clinically diagnosed paucibacillary cases so that such unusual presentations of the disease are treated appropriately and not missed.

## Conclusion

Importantly, the host immune response to *M. leprae *is critical for controlling the infection, but is also responsible for the immunopathological damage that may develop in nerves and specific organ sites. The presentation of a solitary lesion in multibacillary cases remains rare and the onset of a reversal reaction with fast removal of the bacterial load in the lesion further reinforces the view that certain aspects of the host cell-mediated response and pathophysiology of this important disease are still not fully understood. It is important that patients are classified correctly in the leprosy spectrum, so that they may receive the most appropriate treatment.

## Abbreviations

BI: Bacterial index; BT: borderline tuberculoid; IFN-γ: interferon gamma; MB: multibacillary; MDT: multidrug treatment; MLSA-LAM: *Mycobacterium leprae *soluble antigen devoid of lipoarabinomannan; MLCwA: *Mycobacterium leprae *cell wall antigen; MTB PPD: *Mycobacterium tuberculosis *purified protein derivative; PB: paucibacillary; PGL-I: Phenolic glycolipid-I; TT: tuberculoid leprosy; VMT/ST: volunteer muscle testing and sensory test

## Competing interests

The authors declare that they have no competing interests.

## Authors' contributions

Case conceived and analyzed by BRS, KDN and RKM. Laboratory investigations were performed by BRS and KDN. Patient care was done by RKM and follow up of the patient was done by BRS, KDN and RKM. BRS, KDN and RKM discussed the data and BRS prepared the manuscript.

## Consent

Written informed consent was obtained from the patient for publication of this case report and any accompanying images. A copy of the written consent is available for review by the Editor-in-Chief of this journal.
